# Extremely low *Plasmodium* prevalence in wild plovers and coursers from Cape Verde and Madagascar

**DOI:** 10.1186/s12936-017-1892-y

**Published:** 2017-06-08

**Authors:** Josué Martínez-de la Puente, Luke J. Eberhart-Phillips, M. Cristina Carmona-Isunza, Sama Zefania, María José Navarro, Oliver Kruger, Joseph Ivan Hoffman, Tamás Székely, Jordi Figuerola

**Affiliations:** 1 Estación Biológica de Doñana (EBD-CSIC), Américo Vespucio, 26, Seville, E-41092 Spain; 2 CIBER Epidemiología y Salud Pública (CIBERESP), Seville, Spain; 3 Department of Animal Behaviour, Bielefeld University, 33501 Bielefeld, Germany; 4 Department of Biology and Biochemistry, University of Bath, Claverton Down, Bath, BA2 7AY UK; 5 Institut Supérieur de Technologie de Menabe, Morondava, Madagascar; 6 Maio Biodiversity Foundation, Maio, Cape Verde; 7 Wissenschaftskolleg zu Berlin, Wallotstrasse 19, D-14193 Berlin, Germany

**Keywords:** Avian malaria, *Plasmodium*, *Haemoproteus*, *Leucocytozoon*, Wild birds

## Abstract

**Background:**

Relatively little is known about the prevalence of blood parasites in shorebirds, especially those breeding in the tropics. The prevalence of blood parasites of the genera *Plasmodium*, *Haemoproteus* and *Leucocytozoon* was assessed in blood samples from Kentish plovers and cream-coloured coursers in Cape Verde, and samples of Kittlitz’s plovers, Madagascar plovers and white-fronted plovers in Madagascar.

**Results:**

Only two of these samples were positive for *Plasmodium:* a Kittlitz’s plover was infected by a generalist lineage of *Plasmodium* that has already been reported in Europe and Africa, while in a white-fronted plover direct sequencing revealed a previously un-described *Plasmodium* lineage.

**Conclusion:**

Potential explanations for the low prevalence of blood parasites include the scarcity of vectors in habitats used by these bird species and their resistance to parasitic infections.

## Background

Avian malarial parasites of the genus *Plasmodium* and the related *Haemoproteus* and *Leucocytozoon* are vector-borne parasites with often major deleterious effects on birds [1, 2]. Studies based on morphological characterization of blood smears have allowed the identification of diverse blood parasites infecting birds [3]. However, molecular approaches, such as amplifying a fragment of the cytochrome b (*cyt b*) gene, have provided far more detailed insights [4]. These approaches provide greater sensitivity for detecting parasites [5, 6], but see [7] ] and thus allow more accurate and detailed measurement of specificity [4, 8] and the geographical distribution [9] of blood parasites.

The number of studies identifying blood parasites infecting wild birds has increased over the past decade. However, there is still a lack of information regarding parasites that circulate in remote areas or which infect particular wild bird species, such as plovers (*Charadrius* spp.) and coursers (*Cursorius* spp.), which are diverse and have a broad geographical distribution. Previous studies have shown that plovers often carry very few blood parasites, although these studies have mainly focused on migratory species breeding at medium–high latitudes in the northern hemisphere. For instance, Figuerola et al. [10] did not find any blood parasites in smears of Kentish plovers *Charadrius alexandrinus* and Mendes et al. [11] found evidence of blood parasite infection in only 7 of 56 (12.5%) individuals of four *Charadrius* species. Furthermore, in the “Host-parasite catalogue of the avian haematozoa” Bennett et al. [12] reported that only some of the plover species tested (e.g. Kentish plovers, little ringed plover *Charadrius dubius* and three-banded plovers *Charadrius tricollaris*) harbored blood parasites. Moreover, precise information on the parasite lineages infecting these birds is very limited with few *Plasmodium* and *Haemoproteus* lineages infecting *Charadrius* spp. (e.g. [13]; see also the MalAvi database [14]). Therefore, there is a general lack of information on the prevalence and diversity of blood parasite lineages infecting plovers, especially in tropical areas where most of these birds are year-round residents and vector-borne parasite transmission may be favoured. Similarly, *Haemoproteus* and *Plasmodium* parasites have been found in *Cursorius temminckii*, while negative results have been reported in four other *Cursorius* species [12].

Here, the presence of blood parasites belonging to the genera *Plasmodium*, *Haemoproteus* and *Leucocytozoon* infecting plovers and coursers from Cape Verde and Madagascar was molecularly screened. Specifically, this study was focused on four species of the family Charadriidae: the Kentish plover *Charadrius alexandrinus*, the Kittlitz’s plover *Charadrius pecuarius,* the white-fronted plover *Charadrius marginatus* and the endangered Madagascar plover *Charadrius thoracicus*, as well as the related cream-coloured courser *Cursorius cursor* (family Glareolidae).

## Methods

Kentish plovers and cream-coloured coursers were captured between September and December of 2015 at the island of Maio, Cape Verde (15°08′N, 23°13′W). Here, approximately 100–150 pairs of plovers and 20-40 pairs of coursers breed in the sand dunes, salt marshes and semi-desert areas in Salina Porto Ingles, Maio. White-fronted plovers, Kittlitz’s plovers and Madagascar plovers were captured between March and June 2013, 2014 and 2015 in Andavadoaka, Madagascar (−22°05′S, 43°15′E) where they breed on the shoreline of alkaline lakes. In both sites, adults were captured on the nest using funnel traps. Approximately 25–50 μL of blood were collected from the brachial vein of birds and stored in ethanol (96%). See [15] for further details on the field procedures used.

Genomic DNA was extracted using a standard phenol–chloroform protocol. DNA concentration was quantified using both agarose gels and the NanoDrop^®^ ND-1000 Spectrophotometer (Nano Drop Technologies Inc., Wilmington, DE, USA). Samples were diluted using sterile water to a working DNA concentration 20–25 ng/µL. DNA samples from 43 birds were molecularly sexed to check for high quality DNA extraction and all of these samples provided successful amplifications. The presence of *Plasmodium*, *Haemoproteus* and *Leucocytozoon* parasites was screened using the protocol detailed by [16]. In spite of the potential limitations of the molecular identification of blood parasites in bird blood, especially for the detection of mixed infections [17–19], molecular methods are broadly used as they are sensitive approaches providing exciting opportunities to identify a high diversity of blood parasites [4, 8, 14]. In addition, parasite prevalence of haemosporidian parasites estimated using only PCRs is similar to that estimated by microscopy or when comparing both methods [6, 7].

Samples were tested in three independent reactions to reduce the occurrence of false negatives. Both positive and negative controls (water only) were included in each PCR plate. PCR reactions were resolved on 1.8% agarose gels. Amplicons were sequenced by Macrogen (Macrogen Inc., The Netherlands) and sequences were edited using Sequencher™ v 4.9 (Gene Codes Coep., @ 1991–2009, Ann Arbor, MI 48108). Parasite lineages were identified by comparison with sequences deposited in GenBank (National Center for Biotechnology Information). Additional information of the blood parasites identified was obtained from MalAvi [14].

## Results

A total of 215 samples from 208 adult birds were screened for the presence of blood parasites. Of these samples, seven individuals (three Madagascar plovers, two white-fronted plovers and two Kittlitz’s plovers) were sampled twice at different time points. Only two out of 208 individuals tested positive for *Plasmodium* parasites (Table 1): a Kittlitz’s plover and a white-fronted plover captured in Madagascar in 2014 and 2015 respectively. *Haemoproteus* or *Leucocytozoon* parasites were not detected.

**Table 1:** Number of male and female birds captured in Madagascar and Cape Verde

	Madagascar		Cape Verde		
	Females	Males	Females	Males	
Cream-coloured courser			9	11	20
Kentish plover			10	10	20
Kittlitz’s plover	29^a^	29			58
Madagascar plover	28	27			55
White-fronted plover	28	27^a^			55
Total bird sampled	85	83	19	21	208

Three Madagascar plovers, two white-fronted plovers and two Kittlitz’s plovers were sampled twice along the study period

^a^One individual was infected in both species by *Plasmodium*

The *Plasmodium* lineages identified in this study corresponded to a new lineage CHAMAR01 (Genbank accession number: KY053456) isolated from the white-fronted plover and the P_MILAN06 lineage isolated from a Kittlitz’s plover. The lineage CHAMAR01 was amplified in three independent PCR reactions. Amplicons from two independent PCR reactions of this sample were sequenced in both directions obtaining identical results. By comparing this sequence with those deposited in Genbank (fragments of a similar length obtained following the same procedure used here), a 99% overlap was found with sequences P_AFR110 from *Parus griseiventris* from Africa [20] and KOKAKO01 from *Callaeas cinerea* from New Zealand [21]. By contrast, the lineage P_MILAN06 was only amplified once, suggesting the possibility that the bird suffered a low intensity of infection (low concentration of parasite DNA). P_MILAN06 was previously found infecting black kites *Milvus migrans* (Accipitridae; HF543656) and blue-spotted wood doves *Turtur afer* (Columbidae, unpublished according to MalAvi).

## Discussion

Here, the prevalence and diversity of blood parasites infecting four species of plovers and one courser species from Cape Verde and Madagascar was studied. Only two out of 208 individuals were infected by *Plasmodium* parasites (overall prevalence 0.96%). Only a handful of studies have investigated blood parasites infecting wild birds in these areas. For the case of Cape Verde, Hille et al. [22] investigated the blood parasite prevalence in common kestrels using blood smears. During the last years, three studies have identified molecularly the prevalence and diversity of blood parasite lineages infecting wild birds from Madagascar [23–25], adding valuable information to the previous knowledge based on blood smears [26, 27]. Although combining the identification of blood parasites based on the screening of blood smears and molecular approaches could add valuable information [7], molecular methods are broadly used as they allow the identification of genetic diversity and host-range of blood parasites [4, 8, 9, 14, 24]. Furthermore, the prevalence of infection estimated using molecular methods is similar than those measured using smears or combining both microscopy and PCR methods [7]. Nevertheless, further studies are necessary to link the genetic lineages of the parasites found here with their morphological species. The blood parasite prevalence found here was similar to those reported in another species studied in Cape Verde (1.5% in endemic kestrels [22]), but much lower than those reported for other avian species in Madagascar [26]. However, contrary to the case of *Haemoproteus* and *Leucocytozoon* that infected 17.4 and 9.4% of birds in Madagascar, the prevalence of *Plasmodium* found here was similar to that previously reported (0.96% vs 1.9% [26]).

Different factors may explain the low prevalence of blood parasites in this study, for example, resistance to parasite infections in these bird species [28] and/or environmental factors reducing the abundance of the arthropod vectors and consequently limiting the transmission of blood parasites [29]. All plover species including the courser were breeding along salt marshes, thus reducing their exposure to the vectors required for parasite transmission. Thus, a low exposure to insect vectors may partially explain our results, especially those from the Cape Verde archipelago [22]. In fact, the scarcity of potential vectors has been repeatedly suggested as a potential reason explaining the absent or low prevalence of blood parasites found in birds in Macaronesia [22, 30, 31]. Contrary to the case of Cape Verde, Madagascar has a diversity of habitats suitable for vector development, which may explain the blood parasite transmission in this area [26]. It is also possible that the low prevalence found in the plovers was due to resistance to Haematozoa infections, as a low prevalence of infection by blood parasites is usually found in plovers captured in other geographical areas [10, 11].

To our knowledge, only a single study has investigated the potential mosquito vectors of avian *Plasmodium* in Madagascar [24]. *Plasmodium* DNA was found in the head-thorax of mosquito species belonging to the genera *Uranotaenia*, *Culex* and *Anopheles* [24]. In a recent checklist of the mosquitoes in Madagascar, authors recorded additional mosquito genera which could be involved in avian malaria transmission including *Mansonia* and *Coquillettidia* [32] because these genera of mosquitoes have been found involved in the transmission of avian *Plasmodium* in other areas of the world [33]. Although molecular identification of blood parasites in mosquitoes does not imply vector competence, this approach allows a first filter to identify the potential species involved in *Plasmodium* transmission. In this respect, mosquito species belonging to these genera could be involved in the transmission of the *Plasmodium* lineages found here, as *Plasmodium* parasites are considered generalist parasites being transmitted by a number of mosquito species [34, 35].

In addition to the lineage CHAMAR01 described here for the first time, the lineage P_MILAN06 was found infecting a Kittlitz’s plover. Previous information on the occurrence of this parasite lineage in birds provides some information on its geographical distribution and host range. This lineage P_MILAN06 was isolated from an adult black kite captured in Spain [31] and from the resident wood dove in Gabon. This suggests that P_MILAN06 is a generalist parasite lineage that infects species from a variety of orders across a wide geographical distribution that includes, at least, Europe, continental Africa and, probably, Madagascar. However, because this parasite was only amplified in a single PCR reaction, this suggests that this parasite lineage had a low intensity of infection in our study. Further research will be necessary to determine which avian and mosquito species play a key role as reservoirs and vectors of these parasite lineages. Such future studies are plausible as parasites can be detected molecularly in spite of an extremely low intensity of infection in avian blood [8].

## Conclusion

A very low prevalence of infection is found in plovers and coursers from Cape Verde and Madagascar, which could be due to the scarcity of vectors in habitats used by these bird species and their resistance to parasitic infections.
